# Prognostic Impact of Cancer in Patients Hospitalized for Acute Myocardial Infarction: A Population-Based Cohort Study

**DOI:** 10.3390/jcm15103730

**Published:** 2026-05-12

**Authors:** Nicola Cosentino, Filippo Trombara, Matteo Franchi, Daniela Cardinale, Alice Bonomi, Mattia Dall’Asta, Olivia Leoni, Riccardo Maragna, Francesco Cannata, Gianluca Pontone, Giancarlo Marenzi

**Affiliations:** 1 Department of Perioperative Cardiology and Cardiovascular Imaging, Centro Cardiologico Monzino IRCCS, 20138 Milan, Italygianluca.pontone@ccfm.it (G.P.); 2 Centro Cardiologico Monzino IRCCS, 20138 Milan, Italy; filippo.trombara@ccfm.it (F.T.); matteo.franchi@unimib.it (M.F.);; 3 Unit of Biostatistics, Epidemiology and Public Health, Department of Statistics and Quantitative Methods, University of Milano-Bicocca, 20900 Milan, Italy; 4 Cardioncology Unit, Cardioncology and Second Opinion Division, European Institute of Oncology, I.R.C.C.S., 20141 Milan, Italy; 5 Regional Epidemiological Observatory, Lombardy Region, 20138 Milan, Italy; 6 Department of Biomedical, Surgical and Dental Sciences, University of Milan, 20122 Milan, Italy

**Keywords:** acute myocardial infarction, cancer, prognosis, in-hospital mortality, one-year mortality

## Abstract

**Background**: Cancer is common among patients with acute myocardial infarction (AMI) and may influence management and outcomes. The prognostic impact of cancer status (active vs. past) and its anatomical site remains insufficiently defined. We evaluated the association between cancer and short- and long-term outcomes after AMI in a large population cohort. **Methods**: Using linked administrative databases from Lombardy, Italy, we identified adults with a first AMI hospitalization from 2014 to 2022 (N = 124,403). Patients were categorized by cancer history, cancer status (active vs. past), and cancer site. The primary endpoint was in-hospital mortality; secondary endpoints were 1-year all-cause mortality and 1-year rehospitalization for AMI or acute heart failure (AHF). Multivariable log-binomial, Cox, and Fine&Gray models were applied. **Results**: Overall, 18,463 (14.8%) had a history of cancer. They were older and had higher comorbidity burden. Cancer history was associated with higher in-hospital mortality (adjusted risk ratio [RR] 1.06, 95% CI 0.99–1.13) and one-year mortality (adjusted hazard ratio [HR] 1.46, 95% CI 1.40–1.52). Active cancer carried the greatest risk (in-hospital RR 1.07, 95% CI 1.00–1.15; 1-year HR 1.60, 95% CI 1.53–1.68), whereas past cancer showed no excess mortality. Site-specific analyses identified lung (one-year HR 2.69, 95% CI 2.15–3.37) and hematological cancers (one-year HR 2.19, 95% CI 1.88–2.56) as highest-risk. Elevated mortality with cancer was consistent in STEMI and NSTEMI. Competing-risk analyses showed a similar risk of rehospitalization among cancer and non-cancer patients. **Conclusions**: In a real-world, unselected AMI population, cancer worsens short- and long-term survival, especially when active and involving the lungs or the hematopoietic tissues. Incorporating cancer status into AMI risk stratification and strengthening cardio-oncology pathways in acute care are warranted to improve patient outcomes.

## 1. Lay Summary

This study of over 124,000 patients found that having active cancer significantly increases the risk of death following a first heart attack, whereas patients with a history of past cancer that is no longer active have survival rates similar to those without cancer.

## 2. Key Findings

Patients with cancer who suffer a heart attack are less likely to receive intensive heart treatments or recommended medications and face a nearly doubled risk of dying within one year compared to those without cancer.

The impact on survival varies greatly by the type of cancer, with lung and gastrointestinal cancers carrying the highest risks, while breast and prostate cancers show much more modest effects on heart attack outcomes.

## 3. Introduction

Advances in oncology have substantially improved survival across many malignancies, leading to a rapidly expanding population of cancer patients and survivors [1,2]. As a consequence, cardiovascular disease has emerged as a leading non-cancer cause of morbidity and mortality in this population, reflecting the combined effects of shared risk factors, cancer-related systemic inflammation, and treatment-induced cardiotoxicity [3,4,5,6]. Among cardiovascular events, acute myocardial infarction (AMI) represents a particularly critical condition, requiring prompt diagnosis and timely invasive management, yet posing unique clinical challenges in patients with cancer [6,7]. Patients with cancer who develop AMI differ substantially from the general AMI population. They are typically older, more comorbid, and often exposed to prothrombotic and proinflammatory states that may accelerate atherosclerosis and impair recovery [8]. In addition, concerns regarding bleeding risk, frailty, and competing prognosis frequently lead to less aggressive management strategies, including lower use of revascularization and guideline-recommended pharmacotherapies [9,10]. These factors collectively contribute to worse clinical outcomes, as consistently reported in prior studies [3,11,12,13,14].

However, current evidence remains limited in several key aspects. Most available studies have treated cancer as a homogeneous condition, without adequately accounting for the heterogeneity in cancer activity (active vs. past disease) or the marked differences across tumor types [15,16]. These distinctions are clinically crucial, as cancer biology, stage, treatment exposure, and systemic effects vary widely and may differentially influence cardiovascular risk and outcomes. Moreover, large-scale, real-world, population-based analyses addressing both cancer status and anatomical site in the setting of AMI are scarce.

We conducted a retrospective population-based cohort study in the Lombardy region of Italy to assess the prognostic implications of cancer among patients hospitalized for a first AMI. Specifically, we examined whether a history of cancer, its status (active vs. past), and its anatomical site influenced in-hospital mortality, one-year all-cause mortality, and one-year rehospitalization for AMI or acute heart failure (AHF).

## 4. Methods

### 4.1. Data Source

The present study used linkable administrative health databases of the Lombardy region in Italy, which include a population registry with demographic data of all residents and detailed information on hospital records and drug prescriptions. Data are available for about 10 million registered inhabitants of Lombardy from 2004 to 2023. Healthcare in Italy is publicly funded for all residents, irrespective of social class or employment, and everyone is assigned a personal identification number kept in the National Civil Registration System. All registered residents are assisted by general practitioners and are covered by the National Health System (NHS) with high levels of completeness regarding drug prescriptions, diagnosis, and length of observation. The pharmacy prescription database contains the medication name, anatomic therapeutic chemical classification code (ATC), and date of dispensation of drugs reimbursed by the NHS. The hospital database contains information on date of admission, discharge, death, primary diagnosis, and up to five co-existing clinical conditions and procedures performed during the hospitalization, regardless of the payer (NHS, insurance, or out-of-pocket). The diagnoses, uniformly coded according to the 9th International Code of Diseases (ICD-9-CM; Supplementary Table S1) and standardized for all Italian hospitals, are compiled by the hospital specialists directly in charge of the patients and are validated by hospitals against detailed clinical-instrumental data as they determine reimbursement from the NHS or any other payer. A unique identification code allows the linkage of all databases. To ensure individual data protection, each identification code was automatically converted into an anonymous code before we received the dataset. ICD-9-CM diagnostic codes and ATC drug codes used in the current study are reported in Supplementary Table S1.

### 4.2. Study Population

We identified all adult patients (≥18 years) hospitalized between 1 January 2014 and 31 December 2022 with a primary diagnosis of AMI (ICD-9-CM code 410). Only the first hospitalization per patient was considered (index admission). Secondary AMI diagnoses were not considered in the analysis. Patients were excluded if they were not residents of Lombardy in the year of admission or if they had not been continuously registered in the regional health system for at least 10 years prior. Patients were grouped according to history of cancer, including those with past or active cancer. Cancer records were extracted using available administrative data [ICD-9-CM codes, pharmacy prescription database (past outpatient chemotherapy services), and the NHS care co-payment exemption codes]. We defined past cancer as the presence of a cancer diagnosis, cancer-related medications, or an active co-exemption code between 5 and 10 years prior to the index hospitalization for AMI, but not within the 5 years preceding the admission. Active cancer was defined as the presence of a cancer diagnosis, cancer-related medications, or an active co-exemption within the 5 years preceding the index hospitalization for AMI, regardless of the time of cancer onset. Patients with a record of any cancer were also classified by cancer site (five most common cancers: hematological, lung, gastrointestinal, prostate and breast).

### 4.3. Study Variables

The history of comorbidities of interest were retrieved using hospital (including up to six co-existing diagnoses and procedures) and out-of-hospital medical records. Comorbid conditions (hypertension, diabetes, ischemic heart disease, atrial fibrillation, chronic kidney disease, chronic obstructive pulmonary disease) were identified in the 2 years preceding admission. Cardiovascular medications were assessed both in the 2 years before and during the year after hospitalization, including angiotensin-converting enzyme inhibitors/angiotensin receptor blockers, beta-blockers, diuretics, calcium antagonists, lipid-lowering drugs, antiplatelets, and oral anticoagulants. Ward of hospitalization was also evaluated.

### 4.4. Study Outcomes

The primary endpoint of the study was in-hospital mortality. One-year post-discharge all-cause mortality and rehospitalization for AMI/AHF were considered as secondary endpoints. Moreover, in-hospital and 1-year bleeding complications were retrieved. Patients were followed up from the index discharge date until the outcome onset, migration, or up to the end of the one-year follow-up.

### 4.5. Statistical Analysis

Baseline characteristics are summarized using mean and standard deviation for continuous variables, and frequencies with percentages for categorical variables. Between-group differences were assessed by standardized differences. The association between cancer history and in-hospital mortality was evaluated using log-binomial regression, expressed as risk ratios (RRs) with 95% confidence intervals (CIs). One-year mortality was analyzed using Cox proportional hazards regression, and 1-year rehospitalization for AMI/AHF using Fine&Gray competing risk models, with death considered a competing event. Multivariable models were adjusted for the patient characteristics reported in Table 1 and Table 2 that differed between the two groups. Medications after hospital discharge were considered in the models as time-dependent covariates. Differences in cumulative 1-year survival and re-hospitalization for AMI/AHF were plotted using Kaplan–Meier curves according to cancer status. A two-sided *p* value less than 0.05 was required for statistical significance. All analyses were performed using SAS version 9.4 (SAS Institute, Cary, NC, USA).

## 5. Results

### 5.1. Study Population

Between 2014 and 2022, 124,403 patients were hospitalized with a first AMI. Of these, 18,463 (14.8%) had a history of cancer. Baseline clinical characteristics of the population, cardiovascular medications taken before admission and after hospital discharge, and admitting ward for the overall AMI cohort and by cancer history are shown in Table 1. Compared with patients without cancer, those with cancer were older, had a higher comorbidity burden, and were more frequently treated with chronic cardiovascular medications before admission. Patients with cancer were more frequently admitted to Internal Medicine wards, while patients without cancer were more frequently managed in cardiology or intensive care units. Overall, 61.6% of patients underwent percutaneous coronary intervention during the index hospitalization (62.7% in patients without cancer and 53.5% in those with cancer). After discharge, patients with cancer were less likely to receive cardiovascular medications than those without cancer.

### 5.2. Primary and Secondary Endpoints

**Figure 1:**
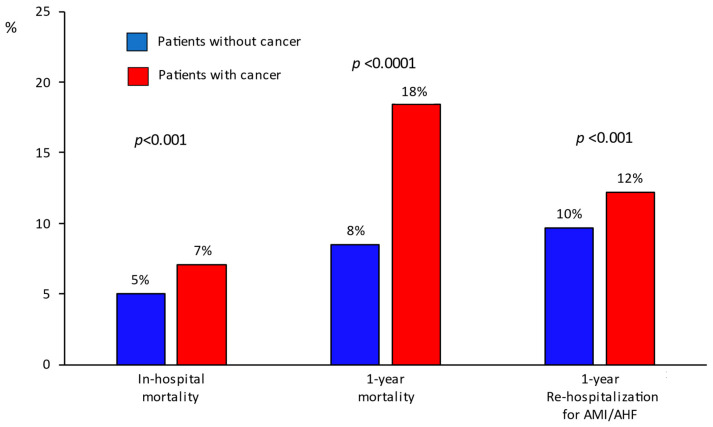
Primary and secondary endpoint rates in the study’s patients grouped according to their history of cancer. AMI/AHF = acute myocardial infarction/acute heart failure.

**Figure 2:**
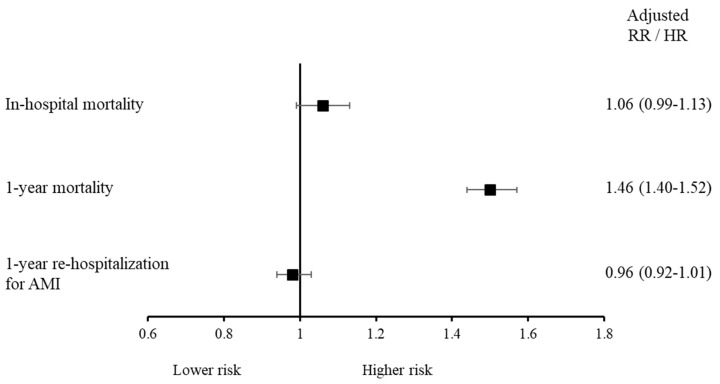
Primary and secondary endpoint risks in the study’s patients grouped according to their history of cancer. AMI/AHF = acute myocardial infarction/acute heart failure; RR = relative risk; HR = hazard ratio.

**Figure 3:**
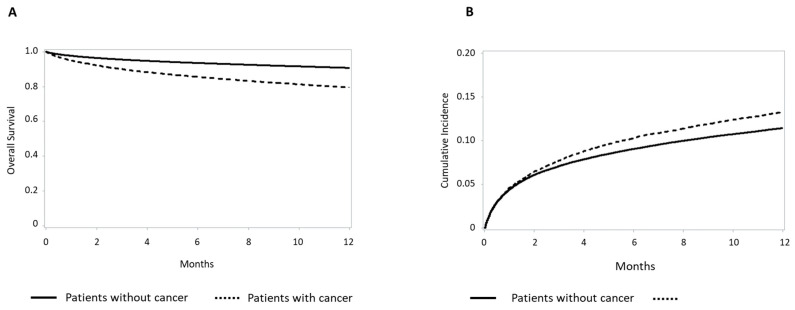
Kaplan–Meier survival (1-year mortality) curves in patients with or without cancer (**A**). Kaplan–Meier curves of rehospitalization for acute myocardial infarction/acute heart failure in patients with or without cancer (**B**).

Primary and secondary endpoint rates and risks in patients grouped according to their history of cancer are shown in Figure 1 and Figure 2. In-hospital mortality was 7.1% among cancer patients and 5.0% in those without cancer, with an adjusted RR of 1.06 (95% CI 0.99–1.13). One-year post-discharge mortality was 18.4% in cancer patients versus 8.5% in non-cancer patients (adjusted HR 1.46, 95% CI 1.40–1.52). Rehospitalization for AMI/AHF occurred in 2085 (12.2%) cancer patients and 9711 (9.7%) non-cancer patients. The adjusted risk for rehospitalization was similar between patients with and without a history of cancer (adjusted HR 0.96, 95% CI 0.92–1.01). Kaplan–Meier analysis showed significantly lower survival in patients with cancer (Figure 3A). Cumulative incidence of rehospitalization for AMI/AHF was also higher in cancer patients, though attenuated after accounting for competing death (Figure 3B). Table 2 reports in-hospital and one-year post-discharge bleeding complications in the study’s patients stratified by cancer history.

### 5.3. Cancer Status and Site

**Figure 4:**
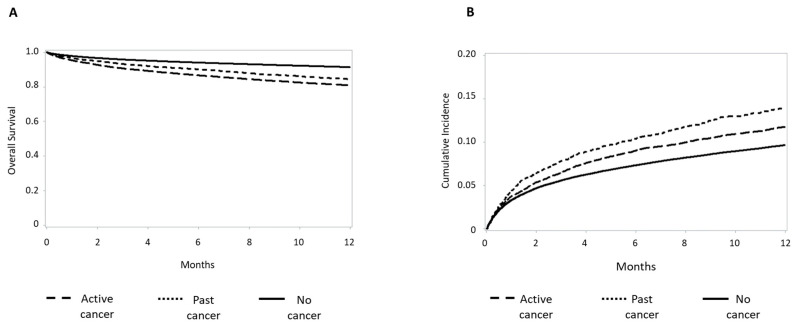
Kaplan–Meier survival (1-year mortality) curves in the study’s patients grouped according to cancer history and status (**A**). Kaplan–Meier curves of rehospitalization for acute myocardial infarction/acute heart failure in the study’s patients grouped according to cancer history and status (**B**).

**Figure 5:**
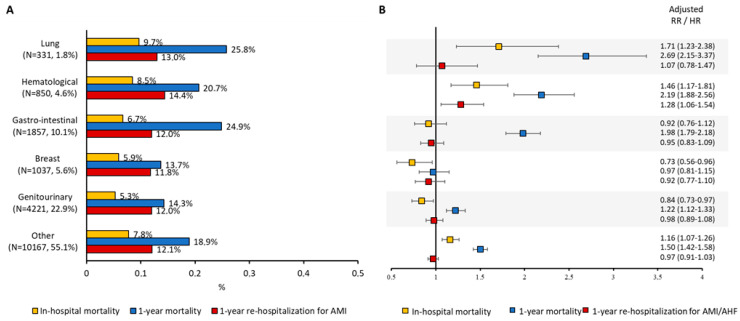
Primary and secondary endpoint rates (**A**) and risks (**B**) according to cancer site. AMI/AHF = acute myocardial infarction/acute heart failure; HR = hazard ratio; RR = relative risk.

Clinical characteristics and study endpoints according to cancer status are reported in Table 3. Overall, 18,463 had a history of cancer, including 14,926 (81%) with active cancer and 3537 (19%) having past cancer. The median time from cancer diagnosis to index AMI hospitalization was 3.9 years (IQR 1.7–6.8) for active cancer and 7.6 years (IQR 6.4–8.9) for past cancer. Patients with active cancer experienced higher in-hospital mortality (RR 1.07, 95% CI 1.00–1.15) and markedly worse one-year survival (HR 1.60, 95% CI 1.53–1.68) compared to patients without cancer history (Table 4). Past cancer was not associated with excess mortality compared to patients without cancer history (Table 3). Adjusted Kaplan–Meier curves for cumulative one-year survival and re-hospitalization for AMI/AHF in patients with past or active cancer are shown in Figure 4A,B, respectively. Table 5 reports in-hospital and one-year post-discharge bleeding complications in the study’s patients stratified by cancer status. Outcomes by cancer site are shown in Figure 5A. Lung and gastrointestinal cancers were associated with the worst prognosis. Lung cancer conferred an adjusted RR of 1.71 (95% CI 1.23–2.38) for in-hospital mortality and an adjusted HR of 2.69 (95% CI 2.15–3.37) for 1-year mortality (Figure 5B). Gastrointestinal and hematologic cancers were also associated with substantially elevated risks. In contrast, breast and genitourinary cancers showed more modest associations.

### 5.4. Outcomes by AMI Type

Among the study cohort, 59,895 patients (48%) presented with ST-segment elevation myocardial infarction (STEMI). The adverse impact of cancer on mortality was consistent across both STEMI and non-ST-segment elevation MI (NSTEMI) subgroups (Table 6). In the STEMI cohort, cancer history was associated with significantly increased one-year mortality (HR 1.61, 95% CI 1.51–1.71). In the NSTEMI cohort, a prior cancer diagnosis was associated with increased in-hospital mortality (RR 1.12, 95% CI 1.00–1.25) and one-year mortality (HR 1.36, 95% CI 1.29–1.44). Across both subgroups, active cancer was consistently associated with the highest mortality risk.

## 6. Discussion

In this large, unselected, population-based cohort, nearly one in nine patients hospitalized for a first AMI had a history of cancer. These patients were generally older, had more comorbidities, and were less frequently admitted to cardiology or intensive care units. They were also less likely to be discharged on guideline-recommended cardiovascular medications. Importantly, cancer was associated with significantly worse outcomes: in-hospital mortality was more than 20% higher, and one-year mortality nearly doubled compared with patients without cancer. The excess mortality associated with cancer was largely attributable to active disease, as outcomes in past cancer survivors approximated those of patients without cancer. The prognostic effect differed substantially by tumor type: lung and gastrointestinal cancers were linked to the highest mortality, while breast and prostate cancers were associated with more modest excess risks. Notably, these patterns were consistent across both STEMI and NSTEMI subgroups, suggesting that the detrimental influence of cancer extends across the full spectrum of AMI.

The co-existence of cancer and AMI is becoming increasingly relevant in clinical practice. Advances in oncologic therapies have improved survival, creating a growing population of patients at risk of cardiovascular disease from both traditional risk factors and treatment-related cardiotoxicity [17,18]. In addition, shared biological pathways—such as systemic inflammation, hypercoagulability, and metabolic dysregulation—predispose to both atherosclerosis and tumor progression. Epidemiologic studies have shown that patients with cancer have a higher incidence of AMI than the general population, and that AMI itself can complicate cancer treatment and prognosis [19,20,21]. This bidirectional relationship underscores the need for integrated cardio-oncologic care and highlights the clinical importance of understanding how cancer modifies outcomes after AMI.

Our findings are in line with and extend prior evidence. Registry-based studies from other countries have consistently reported higher short- and long-term mortality in cancer patients admitted with AMI [5,22]. For example, analyses from the Swedish SWEDEHEART registry [22], the U.S. National Inpatient Sample [16], and the ACTION Registry have shown higher in-hospital mortality and lower use of invasive strategies among patients with cancer. However, most prior studies have treated cancer as a single, homogeneous condition. Few investigations have distinguished active from past cancer, and even fewer have examined site-specific prognostic differences. Our study offers a more detailed perspective, clarifying that prognosis is determined not merely by the presence of cancer but by its clinical activity and anatomical site. In particular, the strikingly elevated risk associated with lung and gastrointestinal cancers underscores the heterogeneity of cancer’s impact on cardiovascular outcomes. In contrast, the absence of excess mortality among cancer survivors suggests that once patients are disease-free and beyond the period of active oncologic treatment, their cardiovascular outcomes after AMI are comparable to those of individuals without cancer. Given the expanding population of cancer survivors worldwide, understanding how remission status modifies cardiovascular prognosis is essential for the long-term management of oncologic patients.

When compared with the existing literature, our study confirms and extends prior findings in several important ways. First, the magnitude of excess risk associated with cancer was consistent with that reported in previous registries, supporting external validity. Second, explicitly distinguishing active and past cancer provides new insights on the prognostic relevance of cancer status. Third, analysis by tumor site identifies the malignancies with the greatest impact on prognosis, yielding insight into site-specific risk. Finally, by including both STEMI and NSTEMI, our study shows that the adverse impact of cancer applies across the spectrum of AMI presentations, suggesting that the underlying mechanisms are generalizable rather than phenotype-specific. Thus, this study moves beyond the traditional binary classification of cancer by simultaneously evaluating cancer activity (active vs. past) and tumor site, providing a more precise and clinically relevant stratification of risk in patients with acute myocardial infarction. By leveraging a large, real-world population-based cohort, the manuscript offers novel insights into the heterogeneity of cancer-related cardiovascular risk, laying the foundation for more personalized management strategies and future cardio-oncology research.

Several mechanisms may explain these results. Clinically, patients with active cancer frequently receive less aggressive management for AMI. Invasive procedures such as coronary angiography and revascularization are often delayed or withheld due to concerns about bleeding risk, frailty, limited life expectancy, or potential interactions with ongoing cancer therapy. Similarly, DAPT or oral anticoagulants may be underprescribed because of perceived or real bleeding risks. This therapeutic nihilism, although often well-intentioned, may contribute to poorer outcomes. From a biological standpoint, cancer induces a systemic prothrombotic and proinflammatory state that accelerates atherosclerosis, promotes plaque instability, and impairs vascular healing. Cancer-related cachexia, renal dysfunction, and hematologic abnormalities may reduce resilience to ischemic injury and impair recovery after AMI. In addition, many anticancer therapies—including platinum-based chemotherapy, fluoropyrimidines, tyrosine kinase inhibitors, and immune checkpoint inhibitors—have direct cardiotoxic effects that may exacerbate ischemic damage or predispose to arrhythmias and heart failure [23,24,25]. The site-specific differences observed in our study are also biologically plausible. Lung and gastrointestinal cancers are frequently diagnosed at advanced stages, often accompanied by systemic inflammation and managed with intensive regimens, all of which may further increase cardiovascular risk. In addition, both cancer types share numerous risk factors that contribute to both tumor development and cardiovascular disease [26]. Finally, in our study, the presence of gastric cancer was associated with an increased risk of hemorrhagic complications during antithrombotic therapy, underscoring the need for careful risk–benefit assessment and close clinical monitoring in patients with gastric cancer requiring antithrombotic treatment.

Our study also carries important implications for health system organization and clinical decision making. Cancer status should be systematically incorporated into AMI risk stratification tools and prognostic models. Recognizing that active cancer substantially modifies both short- and long-term outcomes could inform decisions about the intensity of invasive management and guide patient and family discussions about prognosis. In addition, the strong association between cancer and reduced prescription of evidence-based secondary prevention therapies highlights a treatment gap that may be modifiable. Integration of cardio-oncology services into acute cardiovascular care could help bridge this gap, balancing ischemic and bleeding risks, tailoring reperfusion strategies, and ensuring that cancer patients receive optimal cardiovascular protection.

The strengths of our study include its large sample size, population-based design, and comprehensive linkage of administrative databases that captured hospitalizations, prescriptions, and outpatient care. This allowed for robust outcome assessment across a real-world, unselected cohort. However, limitations must also be acknowledged. The reliance on administrative data means that misclassification of diagnoses is possible, and we lacked some clinical variables reflecting infarct size, coronary anatomy, and left ventricular function. Information on cancer stage, treatment modality, and cause of death was unavailable, preventing more detailed mechanistic exploration. Although we adjusted for major confounders, residual confounding cannot be excluded. Moreover, given the extended study period, it should be acknowledged that contemporary management of AMI evolved over time, reflecting updates in international guidelines and the progressive adoption of novel pharmacological and interventional strategies, which may have influenced treatment patterns and clinical outcomes. Finally, our findings are derived from a single Italian region and require validation in other populations with different healthcare systems.

In conclusion, cancer is a frequent comorbidity among patients hospitalized with AMI and is independently associated with worse in-hospital and one-year survival. The excess risk is driven primarily by active disease and is most evident in patients with lung or gastrointestinal cancers. These findings refine the current understanding of the relationship between cancer and AMI, moving beyond a binary classification of cancer status, and highlight the need for integrated cardio-oncology expertise in acute care. Future research should explore strategies to optimize AMI management in cancer patients, balancing the benefits of invasive and pharmacologic therapies against bleeding and treatment-related risks, while tailoring approaches to cancer type and activity.

## Figures and Tables

**Table 1:** Characteristics of patients hospitalized for acute myocardial infarction between 2009 and 2022 according to cancer history.

	Overall N = 124,403	Patients w/o Cancer N = 105,940	Patients w/Cancer N = 18,463	Standardized Difference *
**Age (years)**	70.1 ± 13.3	69.1 ± 13.5	75.8 ± 10.2	−0.56
**Gender (males)**	83,385 (67.0)	70,767 (66.8)	12,618 (68.3)	−0.03
**History of comorbidities, n (%)**				
Hypertension	23,794 (19.3)	20,013 (18.9)	3781 (20.5)	−0.04
Diabetes mellitus	14,583 (11.7)	12,113 (11.4)	2470 (13.4)	−0.06
Chronic IHD	29,332 (23.6)	24,939 (23.5)	4393 (23.8)	−0.01
Atrial fibrillation	11,915 (9.6)	9560 (9.0)	2355 (12.8)	−0.12
Chronic renal disease	7499 (6.0)	5779 (5.5)	1720 (9.3)	−0.15
COPD	4063 (3.3)	3163 (3.0)	900 (4.9)	−0.10
**Number of comorbidities, n (%)**				
0	64,289 (51.7)	55,562 (52.5)	8727 (47.3)	0.10
1	37,716 (30.3)	32,084 (30.3)	5632 (30.5)	0.00
2	15,497 (12.5)	12,793 (12.1)	2704 (14.7)	−0.08
3	5407 (4.4)	4331 (4.1)	1076 (5.8)	−0.08
>3	1494 (1.2)	1170 (1.1)	324 (1.8)	−0.05
**Medications of interest**				
**Before hospitalization, n (%)**				
ACE-I/ARBS	66,318 (53.3)	54,841 (51.8)	11,477 (62.2)	−0.21
Beta-blockers	44,180 (35.5)	35,942 (33.9)	8238 (44.6)	−0.22
Diuretics	47,221 (38.0)	38,237 (36.1)	8984 (48.7)	−0.26
Ca-antagonists	37,722 (30.3)	30,631 (28.9)	7091 (38.4)	−0.20
Lipid-lowering drugs	45,905 (36.9)	37,668 (35.6)	8237 (44.6)	−0.19
Antiplatelet drugs	41,875 (33.7)	33,656 (31.8)	8219 (44.5)	−0.26
Oral anticoagulant drugs	9513 (7.7)	7329 (6.9)	2184 (11.8)	−0.17
**Procedure during hospitalization, n (%)**				
Percutaneous coronary intervention	84,919 (68.3)	73,665 (69.5)	11,254 (61.0)	0.18
**After hospital discharge, n (%)**				
ACE-I/ARB	80,487 (64.7)	69,747 (65.8)	10,740 (58.2)	0.16
Beta-blockers	91,612 (73.6)	78,887 (74.5)	12,725 (68.9)	0.12
Diuretics	49,795 (40.0)	41,279 (39.0)	8516 (46.1)	−0.15
Ca-antagonists	28,002 (22.5)	23,585 (22.3)	4417 (23.9)	−0.04
Lipid-lowering drugs	100,671 (80.9)	87,101 (82.2)	13,570 (73.5)	0.21
Antiplatelet drugs	104,014 (83.6)	89,700 (84.7)	14,314 (77.5)	0.18
Oral anticoagulant drugs	13,611 (10.9)	11,101 (10.5)	2510 (13.6)	−0.10
**Ward of hospitalization, n (%)**				
Cardiology	34,858 (28.0)	29,508 (27.9)	5350 (29.0)	−0.02
Coronary Unit	70,835 (56.9)	61,277 (57.8)	9558 (51.8)	0.12
Intensive Care Unit	5031 (4.0)	4411 (4.2)	620 (3.4)	0.04
Internal Medicine	7990 (6.4)	6260 (5.9)	1730 (9.4)	−0.13
Other	5689 (4.6)	4484 (4.2)	1205 (6.5)	−0.10

Abbreviations: ACE-I, angiotensin-converting enzyme inhibitor; ARB, angiotensin receptor blocker; COPD, chronic obstructive pulmonary disease; IHD, ischemic heart disease. * Standardized differences < 0.10 may be considered negligible.

**Table 2:** Association between cancer history and bleeding outcomes.

	In-Hospital Bleeding		1-Year Bleeding	
	No. of events (%)	RR (95% CI)	No. of events (%)	HR (95% CI)
Patients without cancer	1162 (1.1)	Ref.	6295 (6.3)	Ref.
Patients with cancer	381 (2.1)	1.44 (1.28–1.62)	1600 (9.3)	1.26 (1.20–1.34)

CI, confidence interval; HR, hazard ratio; RR, relative risk.

**Table 3:** Characteristics of patients hospitalized for acute myocardial infarction between 2009 and 2022 according to cancer history and status (active vs. previous history).

	Patients with Past Cancer N = 3537	Patients with Active Cancer N = 14,926	Standardized Difference
**Age (years)**	77.5 ± 10.4	75.4 ± 10.1	0.20
**Gender (males)**	2302 (65.1)	10,316 (69.1)	−0.09
**History of comorbidities, n(%)**			
Hypertension	712 (20.2)	3069 (20.6)	−0.01
Diabetes mellitus	510 (14.4)	1960 (13.1)	0.04
Chronic IHD	874 (24.7)	3519 (23.6)	0.03
Atrial fibrillation	471 (13.3)	1884 (12.6)	0.02
Chronic renal disease	404 (11.4)	1316 (8.8)	0.09
COPD	168 (4.8)	732 (4.9)	−0.01
**N° of comorbidities, n(%)**			
0	1631 (46.1)	7096 (47.5)	−0.03
1	1051 (29.7)	4581 (30.7)	−0.02
2	562 (15.9)	2142 (14.4)	0.04
3	220 (6.2)	856 (5.7)	0.02
>3	73 (2.1)	251 (1.7)	0.03
**Medications of interest**			
**Before hospitalization, n(%)**			
ACE-I/ARBS	2217 (62.7)	9260 (62.0)	0.01
Beta-blockers	1585 (44.8)	6653 (44.6)	0.00
Diuretics	1758 (49.7)	7226 (48.4)	0.03
Ca-antagonists	1341 (37.9)	5750 (38.5)	−0.01
Lipid-lowering drugs	1584 (44.8)	6653 (44.6)	0.00
Antiplatelet drugs	1657 (46.9)	6562 (44.0)	0.06
Oral anticoagulant drugs	443 (12.5)	1741 (11.7)	0.03
**Procedure during hospitalization, n (%)**			
Percutaneous coronary intervention	2138 (60.5)	9116 (61.1)	−0.01
**After hospital discharge, n(%)**			
ACE-I/ARB	2044 (57.8)	8696 (58.2)	−0.01
Beta-blockers	2390 (67.6)	10,335 (69.2)	−0.04
Diuretics	1729 (48.9)	6787 (45.5)	0.07
Ca-antagonists	874 (24.7)	3543 (23.7)	0.02
Lipid-lowering drugs	2588 (73.2)	10,982 (73.6)	−0.01
Antiplatelet drugs	2760 (78.0)	11,554 (77.4)	0.01
Oral anticoagulant drugs	506 (14.3)	2004 (13.4)	0.03
**Ward of hospitalization, n(%)**			
Cardiology	1037 (29.3)	4313 (28.9)	0.01
Coronary Unit	1852 (52.4)	7706 (51.6)	0.01
Intensive Care Unit	109 (3.1)	511 (3.4)	−0.02
Internal Medicine	361 (10.2)	1369 (9.2)	0.03
Other	178 (5.0)	1027 (6.9)	−0.08

ACE-I, angiotensin-converting enzyme inhibitor; ARB, angiotensin receptor blocker; COPD, chronic obstructive pulmonary disease; IHD, ischemic heart disease. Standardized differences < 0.10 may be considered negligible.

**Table 4:** Association between cancer status and study outcomes.

	In-Hospital Mortality		1-Year Mortality		1-Year Re-Hospitalization for AMI/AHF	
	No. (%) of events	RR (95% CI)	No. (%) of events	HR (95% CI)	No. (%) of events	HR (95% CI)
Patients with past cancer	274 (7.8)	1.01 (0.88–1.15)	504 (15.5)	0.99 (0.91–1.09)	454 (13.9)	1.02 (0.92–1.12)
Patients with active cancer	1040 (7.0)	1.07 (1.00–1.15)	2643 (19.0)	1.60 (1.53–1.68)	1631 (11.8)	0.95 (0.90–1.00)
*p*-value *		0.425		<0.001		0.204

AHF/AMI, acute heart failure/acute myocardial infarction; HR, hazard ratio; RR, relative risk. * *p*-values for the comparison between active and past cancer.

**Table 5:** Association between cancer status and bleeding outcomes.

	In-Hospital Bleeding		1-Year Bleeding	
	No. of events (%)	RR (95% CI)	No. of events (%)	HR (95% CI)
Patients with past cancer	55 (1.6)	1.02 (0.78–1.35)	298 (3.8)	1.10 (0.98–1.23)
Patients with active cancer	326 (2.2)	1.54 (1.36–1.75)	1302 (9.4)	1.31 (1.23–1.39)
*p*-value		0.005		0.078

Abbreviations: CI, confidence interval; HR, hazard ratio; RR, relative risk.

**Table 6:** Study outcomes according to STEMI and NSTEMI subgroups.

	In-Hospital Mortality	1-Year Mortality	1-Year Re-Hospitalization for AMI/AHF
	RR (95% CI)	HR (95% CI)	HR (95% CI)
STEMI			
Cancer vs. no cancer	1.02 (0.94–1.11)	1.61 (1.51–1.71)	0.89 (0.83–0.97)
Active cancer vs. no cancer	1.02 (0.93–1.13)	1.72 (1.61–1.84)	0.89 (0.81–0.97)
Past cancer vs. no cancer	1.02 (0.85–1.21)	1.16 (1.00–1.34)	0.92 (0.77–1.08)
*p*-value *	0.934	<0.001	0.674
NSTEMI			
Cancer vs. no cancer	1.12 (1.00–1.25)	1.36 (1.29–1.44)	1.01 (0.95–1.07)
Active cancer vs. no cancer	1.16 (1.03–1.31)	1.51 (1.43–1.60)	0.99 (0.93–1.06)
Past cancer vs. no cancer	0.99 (0.80–1.24)	0.91 (0.81–1.02)	1.07 (0.95–1.21)
*p*-value *	0.224	<0.001	0.218

* *p*-values for the comparison between active and past cancer. Abbreviations: AHF/AMI, acute heart failure/acute myocardial infarction; RR, relative risk; HR, hazard ratio; STEMI, ST-elevation myocardial infarction; NSTEMI, non-ST-elevation myocardial infarction.

## Data Availability

Data belong to the Lombardy Region and may be available upon request.

## Supplementary Materials

The following supporting information can be downloaded at https://www.mdpi.com/article/10.3390/jcm15103730/s1, Table S1: ICD-9-CM diagnostic codes and ATC drug codes used in the current study.
