# Supplementary material for: Prognostic Impact of Cancer in Patients Hospitalized for Acute Myocardial Infarction: A Population-Based Cohort Study
Source: J Clin Med. 2026 May 12;15(10):3730. doi: 10.3390/jcm15103730 (PMC13207830; doi:10.3390/jcm15103730)
Supplement: Supplementary file 1 [file jcm-15-03730-s001.zip › jcm-4269660-supplementary.pdf]

Supplementary Table S1. ICD-9-CM diagnostic codes and ATC drug codes used in the current study.

**Drugs**	**ATC codes**
ACE-I/ARBS	C09A, C09C, C09BA, C09BB, C09DA, C09DB, C09BX01
Beta-blockers	C07A, C07B, C07C, C07D
Diuretics	C03, C07B, C07C, C07D, C07G, C09BA, C09BX01, C09DA
Ca-antagonists	C08C, C08D, C08E, C08G, C09BB, C09BD, C09BX01
Lipid-lowering drugs	C10
Antiplatelet drugs	B01AC
Oral anticoagulant drugs	B01AA, B01AE, B01AF
Antineoplastic drugs	L01
**Drugs**	**Outpatient's service (Regional codes)**
Antineoplastic drugs	99.25, MAC01, MAC02, MAC03, MAC04
**Diseases**	**ICD-9-CM codes**
Myocardial infarction	410
STEMI	410, except 410.71 and 410.72
NSTEMI	410.71, 410.72
Acute heart failure	428
Hypertension	401-405
Diabetes mellitus	250
Chronic IHD	414
Atrial fibrillation	427.31
Chronic renal disease	585
COPD	490-492, 494, 496
Any cancer	140-208, V58.1 (diagnostic codes) and 99.25 (intervention code)
Lung cancer	162.3, 162.4, 162.5, 162.8
Hematological cancer	200-208
Gastro-intestinal cancer	150-159
Breast cancer	174, 175
Genitourinary cancer	179-189
Bleeding	285.1, 456.0, 459.0, 530.82, 531.0, 532.0, 532.2, 533.0, 533.2, 533.4, 533.6, 569.3, 569.85, 578.1, 578.9, 599.7, 627.1, 729.92, 786.3
**Diseases**	**Exemption codes**
Cancer	048

Abbreviations: ACEi, angiotensin-converting enzyme inhibitors; ARB, angiotensin receptor blockers; COPD, chronic obstructive pulmonary disease; IHD, ischaemic heart disease.
